# Faultline configurations affecting the entrepreneurial team performance of new generation of returning migrant workers in China: An empirical study based on fuzzy-set qualitative comparative analysis

**DOI:** 10.3389/fpsyg.2022.918128

**Published:** 2022-10-12

**Authors:** Zhaoxuan Qiu, Keyue Shen, Nargiz Zhanabayeva, Tingting Shan

**Affiliations:** ^1^Business School, Hohai University, Nanjing, China; ^2^School of Management, Nanjing University of Posts and Telecommunications, Nanjing, China

**Keywords:** the new generation of migrant workers, team faultline, fsQCA, returning entrepreneurship, Rural Revitalization Strategy

## Abstract

As the impact of faultlines is still without a consensus, to figure out how faultlines will hurt or promote the entrepreneurial performance can help the new generation of Chinese migrant workers to start their businesses successfully under the Rural Revitalization Strategy. This study addressed a fuzzy-set qualitative comparative analysis (fsQCA) based on 32 returning entrepreneurial teams from a complexity perspective. We firstly introduced three faultline categories for migrant workers and selected five of the faultlines with high factor loads in each category for further analysis. Then a scale was developed to measure the team performance. By conducting fsQCA, four types of faultline configurations were found: (1) background-experience actuation; (2) guidance-balance lacking; (3) role-cognition conflict; and (4) information-decision polarization. The “background-experience actuation” type will promote the entrepreneurial performance while the other types will hurt the performance. Theoretically, breaking through the limitations of traditional regressions in previous studies, fsQCA is used to explore the complex interactions and integrated effects among different categories of faultlines, demonstrates that the unstable impact is just a one-sided representation of the overall effect, and fills the general faultline theory with Chinese specific scenario and small-sized entrepreneurship. Practically, several implications are proposed to optimize the heterogeneity of the returning migrant workers’ entrepreneurial teams and increase their performances, such as constructing the “balance” and “guidance” mechanism, enriching the background diversity of the members and solving the information-decision faultlines into individual diversity, etc., which can also be utilized by migrant worker entrepreneurs in other developing areas in the world.

## Introduction

The construction of modern business in rural areas is always a challenging problem in the world (Malecki, 2003), especially in the developing countries in Southeast Asia, Africa, Latin America, etc., where the rural laborers tend to flow into metropolises with opportunities and resources that cannot be acquired in rural areas, leading to more serious urban-rural imbalance and abnormal urbanization. These workforces are the so-called “migrant workers” who earn their livings in the city while actually have a rural identity. Considering that the extreme outflow of the workforces may contribute to the “the rural penalty” and “rural differential” (Hite, 1997; Hobbs and Blodgett, 1999) restricting the economic development of the countryside, governments across the world are trying to balance the developing level and resource distribution between urban and rural areas, and encourage migrant workers with knowledge and resources to flow back to their rural hometowns. In China, the government has put forward the “Rural Revitalization Strategy,” and taken measures to facilitate economic development, farmers’ employment and entrepreneurship in rural areas by encouraging the integration of the First, Second, and Third Industry (Cpc Central Committee and State Council, 2022). By excavating multiple values in rural areas, cultivating advantageous and characteristic industrial clusters and implementing comprehensive measures such as the “Rural Revitalization through Digital Commerce” project, several progresses will be made in the field of the agricultural modernization, and the “common prosperity” of people will be significantly promoted. Under these initiatives, various supporting policies have been enacted to attract young migrant workers to leave for their hometowns to start their own businesses. Among them, the new generation of migrant workers has gradually become a prominent part. They play an important role in China’s new urbanization and healthy economic and social development (Liu and Xia, 2017), and have become a crucial micro-entity to facilitate the implementation of the Rural Revitalization Strategy and a crucial starting point for policymakers to create equal opportunities between urban and rural areas, inclusive economic growth and shared prosperity.

The new generation of migrant workers in China refers to an emerging group of migrant workers who were born after 1980s, have received some education but have almost no agricultural experience and are engaged in non-agricultural activities in the cities (Wang, 2001). They have not only experienced major economic and social changes such as the comprehensive Reform and Opening-up and the advent of the digital age, forming various background-experience differences, but also received their basic education and job training in the cities and have accumulated a lot of knowledge and experience for basic production, as well as boarder views and richer networking than their rural peers. These factors lead to the great heterogeneity and diversity of Chinese migrant workers in intergenerational, growth background, expertise, and entrepreneurial cognition, which is a basis for bringing rare, unique external commercial resources, information, and knowledge into the entrepreneurial teams (Bourdieu, 1986; Coleman, 1988; Burt, 1997; Acquaah, 2007) so as to enhance their dynamic capability to adjust to the uncertain changes. As the studies of the diversity are gradually deepened from the individual into subgroup level, faultlines proposed by Lau and Murnighan (1998) can be introduced to analyze the diversity among subgroups in their entrepreneurial organizations. To figure out how faultlines will hurt or promote the entrepreneurial performance can help the new generation of Chinese migrant workers to start their businesses successfully under the Rural Revitalization Strategy.

Previous studies mainly divide team faultlines into two categories, i.e., the social-category and information-based faultlines. Most scholars believe that in general, the social-category faultlines tend to negatively affect the entrepreneurial results, while the information-based faultlines tend to positively affect the entrepreneurial results (Bezurukova et al., 2009, Bezrukova et al., 2012; Hutzschenreuter and Horstkotte, 2013; Cooper et al., 2014; Zhang et al., 2020). They supposed that the social-category faultlines will bring the social stereotypes and bias among subgroups and causing “group-in” and “group-out” barriers that damage the communications and performance (Byrne, 1971; Lincoln and Miller, 1979; Jackson et al., 1995), but the information-based faultlines will make the knowledge of the whole team more diverse to finish the group tasks, so as to promote the entrepreneurial performance. There are also some researchers proposing different opinions about the influencing direction of the faultlines. Some of them suppose that the information-based faultlines can act detrimentally (Baek Choi and Thomas, 2009; Georgakakis et al., 2017) while others suggest that the relationship between faultlines and the results is not linear (Sun, 2015; Hu and Ge, 2020; Ma et al., 2021).

Although the effect of the faultlines have been discussed for decades due to its complex mechanism, it is still in discussion without a consensus. By applying traditional research methods such as multiple linear regression, structure equation model (SEM), and moderated mediating model, etc., the existing studies can only focus on the net effect of a single variable (Ragin, 1987, 2000; Ragin and Fiss, 2008), including relationships among the independent and independent variables (*x* to *y*), mediators (*x* to *m* to *y*) and moderators (*x* to *y* under *m*). As the final impact of faultlines may depend on their comprehensive effects (Bezrukova et al., 2012), the linear relationships might not completely reflect the intricate interactions and integrated influence of multiple sorts of faultlines on the entrepreneurial results (e.g., *x*_1_ + *x*_2_ + *x*_3_ to *y*) in the complex managerial scenarios (Misangyi et al., 2017). Therefore, the combinations formed by antecedent conditions in the team, which are the so-called “configurations,” should be introduced for this research (Miller, 1986, 1996).

Based on the configuration perspective, the fuzzy-set qualitative comparison analysis (fsQCA) overcomes the limitations of correlation or regression research methods, and discusses the complex causal relationship between conditional configuration and outcome variables from an overall view (Miller, 1986; Ragin, 2000, 2008; Lacey and Fiss, 2009), and is widely used to explore the complexity problems in the managerial reality (Meyer et al., 1993; Delery and Doty, 1996; Miller, 1996; Ragin, 2000). *Via* conducting the QCA, we can find out the faultline configurations that cause the high or non-high performance of the new generation of returning migrant workers’ entrepreneurial teams and have a better understanding of the various comprehensive effects of different types of demographic faultlines, and carry out investigations on whether the relationship between these faultline configurations and organizational performance has a causal asymmetry (Rihoux and Ragin, 2009), i.e., if configuration *x* can cause the outcome *y*, can we conclude that the configuration *x* must exist when the outcome *y* occurs?

The contributions and novelties in this study contain both theoretical and practical aspects as follows: theoretically, breaking through the limitations of traditional correlations and regressions, fsQCA is used as a new method to explore the complex interactions among different categories of faultlines in the returning entrepreneurial teams and focus on the integrated effects of faultlines, which explains that the unstable impact of the faultlines influenced by role cognition, gender stereotype and balanced interpersonal relationship is a one-sided representation of the overall effect, and fills the general faultline theory by being applied and analyzed in Chinese specific scenario and small-sized entrepreneurship. Practically, cognitive theory is applied to optimize the human resource structure of the returning migrant workers’ entrepreneurial teams and increase their performances, and several suggestions corresponding to each faultline configurations have been proposed, which provides an academic basis for the relevant policy departments to take specific and accurate countermeasures to promote the concrete implementation of the Rural Revitalization Strategy in China, and can also be utilized by migrant worker entrepreneurs planning to start their businesses under similar conditions of member heterogeneity in Southeast Asia, Latin America, Africa, and other developing areas.

This article is organized as follows. Section “Literature review” illustrates a complete review for the disagreement in the impact of faultlines, as well as the mechanism of the three faultline categories or dimensions based on existing literature. Section “Materials and methods” introduces the research design in detail. Section “Results” shows the results of the fsQCA. In section “Discussion” we have a throughout discussion about the theoretical values, managerial implications and suggestions, and the limitations and the future works.

## Literature review

### The impact of team faultlines

The concept of team faultline was put forward to deepen the research on the impact of team diversity on team dynamics (Lau and Murnighan, 1998). The team faultline is a hypothetical boundary of one or more subgroups, which will divide the whole team into several subgroups according to different demographic characteristics. Under the division of team faultline, the members of each subgroup have one or more similar demographic characteristics, forming the homogeneity of members in the subgroup and the heterogeneity between subgroups. The members inside and outside the subgroup are divided into “group-in” and “group-out” members. Inspired by the concept of faultline in geology, this concept emphasizes three comparable characteristics: (1) the characteristics of different dimensions of members in the subgroup are similar to different strata and have a sense of levels; (2) potential faultlines need to be activated by external forces; (3) the strong team faultline will show the importance of different attribute levels between subgroups and increase the possibility of conflicts between subgroups.

The analysis of the impact or effectiveness of the team faultline, i.e., the relationship between the team faultline and the team processes and results, in particular team performance, is the focus of research on the team faultline. Currently, academic community has conducted extensive empirical research on the direct effect of team faultlines on performance and the effect of different types of faultlines on performance through different intermediary and moderating variables from the two perspectives of generality and contingency.

#### General perspective

Early studies focused on the direct negative effects of the team faultlines on team performance from a general perspective. Originally, Lau and Murnighan (1998) proposed that team conflict caused by the demographic attributes of the team faultline would exacerbate mistrust among members and reduce group satisfaction. By analyzing the subgroup fragmentation in the workgroups or the TMTs in real business organizations, many scholars are also convinced that, with the increase of the general faultline strength, the team performance will be spoiled (Li and Hambrick, 2005; Barkema and Shvyrkov, 2007; Ndofor et al., 2015; Vandebeek et al., 2016). However, as the mechanism of the impact of team faultline on an enterprise’s performance may be highly complex, it’s difficult to draw a firm conclusion just by simply considering the correlation between the two. Therefore, the research on the effectiveness of faultline will soon change the perspective of generality into contingency, which is also at the root of the long-standing disagreement about the relationship between team failure and performance, i.e., as the managerial scenario changes, the faultlines might act totally differently.

#### Contingency perspective

In the stage of conducting research from the perspective of contingency, the academic community classifies the team faultline into social-category faultlines, characterized by demographic attributes such as gender, age, and race, and information-related faultlines, characterized by educational background and work experience, according to the correlation between the faultline and team tasks (Bezurukova et al., 2009), or bio-demographic faultlines and task-related faultlines (Hutzschenreuter and Horstkotte, 2013), and discussed in combination with mediation and moderating model. This kind of research refined the independent variable types of faultlines and laid a foundation for exploring the comprehensive action mechanism of various complex types of faultlines. However, the academic community has produced the following main divergent views on the action direction of different types of faultlines:

1. Information-related faultlines are usually positively correlated with team performance, while social-category faultlines are generally negatively correlated with team performance. Bezurukova et al. (2009) put forward this view and studied the team identification and faultline width as moderating variables; later, Bezrukova et al. (2012) further believed that if the information-related faultlines could not offset the negative impact of the social-category faultlines, the team performance would show a downward trend; Through empirical research on 61 German enterprises, Hutzschenreuter and Horstkotte (2013) also verified the positive effect of bio-demographic faultlines on product extension and the negative impact of task-related faultlines on that; Zhang et al. (2020) further argued that the social-category faultlines and information-related faultlines of entrepreneurial team jointly affect the intermediary variables of role clarity under the moderating effect of team identity, and ultimately affect entrepreneurial performance. Cooper et al. (2014) indicated that information-based faultline strength promotes the performance under low environmental dynamism, high complexity, and high munificence, while hurts the performance under high environmental dynamism, low complexity, and low munificence. Tuggle et al. (2010) made a research on the faultlines’ impact on several fields including strategies, innovation, international expansion as well as decision-making, and all of the results show the detrimental effect of the social-category faultlines.
2. The information-related faultlines have a significant negative effect, but the effect of social-category faultlines is unpredictable. For example, Georgakakis et al. (2017) conducted empirical research on 248 large international companies, focusing on the impact of CEO-TMT social-category faultlines and enterprise performance on the company’s financial performance under the adjustment of intermediary variables such as CEO-TMT similarity, tenure overlap, experience diversity and other mediating variables, while the impact of knowledge-related faultlines was not significant; Baek Choi and Thomas (2009) found that both relationship-focused faultlines and task-focused faultlines impair organizational performance.
3. The strength of both information-related and social-category faultlines has an inverted “U” relationship with enterprise performance. For instance, the research of Sun (2015) and Hu and Ge (2020) shows that with the increase of the strength of the two types of team faultlines in the entrepreneurial team, under the moderating effect of various variables, its influence on the team innovation performance changes from negative to positive and then back to negative. Ma et al. (2021) analyzed listed manufacturing corporates in China and found that there is also an inverted U-shaped relationship between task-related faultline and green technology innovation, while bio-demographic faultline has no significant influence on green technology innovation. Only by controlling the strength of team faultlines in a reasonable range can the company’s innovation performance reach the best level.

Besides, scholars also draw their attention to figure out how environmental factors mediating or moderating the impact of the faultlines based on the contingency perspective:

1. For mediating effects, scholars have paid their attentions to the intermediation of the further inter-relationship among members. Quite a few scholars took relationship and task conflict as intermediary variables, and indicated that both information-based and social-category faultlines will possibly raise the relationship and task-focused faultlines, and both of the two conflicts may spoil the team performance (Pearsall et al., 2008; Baek Choi and Thomas, 2009; Thatcher and Patel, 2012); Veltrop et al. (2015) found that the team faultline has the disruptive effects on reflexivity which can promote the team results; Zhang et al. (2020) verified that the role clarity that can strengthen the team performance will be inversely affected by faultlines, etc.
2. For moderating effects, existing research mainly investigated outer factors such as environmental uncertainty (Yang and Zang, 2022), environmental dynamics (Cooper et al., 2014), etc., and inner factors such as team identification (Bezurukova et al., 2009; Zhang et al., 2020), shared objectives (Knippenberg et al., 2010), CEO-TMT interactions and similarities (Kaczmarek et al., 2012; Georgakakis et al., 2017), task interdependence (Kwon and Lee, 2020), dual leadership (Zhang et al., 2019, 2022), etc. Most of the studies we mentioned demonstrate that as the degree of complexity and change of the outside environments increases, the faultlines’ negative effect will be alleviated as the focus of the members will be shifted from inner conflicts to outer survival or development; as the identification and consensus becomes stronger and the communication and learning systems becomes more mature, the overall awareness of members and the paths to deal with conflicts are more complete, so the impact of the faultlines can also be adjusted to positive. These moderating results have challenged the traditional mindset about the negative faultline influence based on general perspective with persuasive empirical evidence.

To sum up, from the perspective of research methods, although the existing studies are of great significance for understanding the mechanism of team faultline effectiveness, most of them use the general and contingency perspective *via* the traditional regression analysis method to study the correlation between the mutually independent faultline conditions and the outcomes to judge the positive and negative effects. Such research based on mutually independent team faultlines is not conducive to considering the impact of multiple team faultlines on team performance comprehensively, as well as the in-depth exploration of the comprehensive mechanism of the complex relationship between different types of team faultlines under managerial practice. This means that a new method which can analyze the integrated impact of different types of the faultlines should be applied in our study to contribute to fill the present research gap.

### Faultline categories in the new generation of Chinese migrant workers’ entrepreneurial teams

Based on the understanding of the new generation of migrant workers and the explanations of the mechanism of different types of team faultlines in previous studies, we summarize three typical categories or dimensions of the faultlines for the new generation of migrant workers’ entrepreneurial teams. Faultlines in the same category have similar natures and mechanism to affect the entrepreneurial performance of the teams. Their detailed influencing mechanisms are as follows.

#### The information-decision faultlines

This type of faultline includes expertise faultline, risk preference faultline, etc. These faultlines are based on the knowledge perspective and preference when making decisions on a specific business behavior in the short-term after starting entrepreneurship, which can be strongly related to the specific business decisions of the returning entrepreneurial team, and affect the formation of the diversified decision-making information resource pool in the entrepreneurial team of the new generation of returning migrant workers. According to information decision theory, when team members are aware of their knowledge differences, they will spontaneously or consciously use the multiple value of differences and efficiently use all available cognitive resources in the team to form a knowledge pool (Tsui et al., 1992; Jehn, 1997; Williams and O’Reilly, 1998; Jehn, 1999; Webber and Donahue, 2001; Bezurukova et al., 2009), and be able to allocate resources in a timely and flexible manner as team members work together to complete tasks and make decisions. Additionally, team members may also be more willing to collaborate across faultlines (Gibson and Vermeulen, 2003; Cramton and Hinds, 2005) to form a “synthetic perspective” based on the whole team, improve the decision-making quality of entrepreneurial teams and lead to the progress of team performance (Schweiger and Sandberg, 1989; Schwenk, 1990; Bezurukova et al., 2009). As the new generation of migrant workers show a low degree of unity between expertise and entrepreneurship and a lack of appropriate support from basic expertise (Shi and Wang, 2020), if their entrepreneurial team has agriculture and industry-related technologies and diversified members with economic and management education or professional experience, it will obviously make their entrepreneurship more scientifically, and enlarge the possibility of their entrepreneurial success. What is more, in terms of making decisions with risks, due to the characteristics of small scales, a high risk, and high proportion of self-raised funds in the entrepreneurship of the new generation of migrant workers, their decision-making is often in contradictory orientations between conservative management and bold innovation. Therefore, the strength comparison of subgroups with different risk preferences will profoundly affect the decision-making tendency of the entrepreneurial teams, resulting in the fluctuations of performance at last.

#### The background-experience faultlines

This type of faultline includes age intergenerational faultline, growth environment faultline, etc. Because of the intergenerational and growth environment differences among the members of the entrepreneurial team of the new generation of migrant workers, they will experience different historical development, information intake, and mindset change in the process of growth, forming different cognition and social skill accumulation, which is a kind of long-term information and cognitive difference. On the one hand, the older generation of migrant workers will use all their resources, such as original capital, social experience, and emotional accumulation (Wang, 2019), to the new generation to fully support or guide the entrepreneurial behavior of the new generation of migrant workers and even directly participate in team entrepreneurship (Liu and Xia, 2017). On the other hand, there has long been a “dual structural” difference between urban and rural areas in China, making the new generation of migrant workers with different growth environments significantly vary: the new generation of migrant workers living or working in cities have more urbanized ideas, behaviors and identity, as well as a broader, diversified vision, and a more abundant social capital and relationship network (Liu and Xia, 2017); while those who grow up in a rural environment are more likely to be exposed to higher life pressures and their personal performance, pressure resistance and adaptability in life experiences are increased, they have stronger entrepreneurial resilience (Connork and Davidson, 2003; Wu et al., 2021). The previously mentioned diversity means that the members of such founding teams, who are influenced by different growth backgrounds, have more complex behavioral intentions and corresponding entrepreneurial behavioral differences (Ajzen, 1991). At the same time, they have the characteristics of adapting to the urban–rural dual economic and social environment, which has a profound impact on their entrepreneurial development.

#### The role-motivation faultlines

This type of faultline includes gender faultline, etc. This type of faultline directly reflects the heterogeneity of returning entrepreneurial team members in gender roles, task roles, and personality roles. Different members will have differentiated motivation and behavior orientation based on their own decision-making. Gender role differences are the most prominent among them: in terms of the choice of entrepreneurial scale, due to the traditional value of “inheritance of the eldest son” in China and the differences in risk preference and income expectation between genders, male returning entrepreneurs prefer to choose large-scale and capital-intense entrepreneurial forms based on the development motivation of “making a big fortune.” Among them, the most important is private enterprise creation and equity investment, which differs greatly from returning women entrepreneurs who choose self-employment and other forms of entrepreneurship based on family survival motivation (Fu et al., 2014). This discrepancy directly affects the return on investment and the absolute return on relevant entrepreneurial projects. When it comes to product and service innovation, women entrepreneurs can give their thinking advantages more space than men, design unique products and services, and reduce the market competition entrepreneurial teams face (Huang et al., 2012). The differences above show that the gender faultline in the entrepreneurial teams of the new generation of returning migrant workers will affect the development size and the competitive level of the team in the market by influencing entrepreneurial motivation and behavior in making relevant decisions.

## Materials and methods

### Methods

The fsQCA was first proposed by Ragin (1987). Based on the configuration perspective, the organization is best understood as a cluster of interconnected structures and practices rather than a single entity or loosely combined entity, so it cannot be understood in terms of an isolated analysis of components (Fiss, 2007). It overcomes the limitations of correlation or regression research methods on the “net effect” of independent variables on dependent variables (Rihoux and Ragin, 2009). It discusses the complex causal relationship between conditional configuration and result variables from an overall perspective, which is closer to the actual management situation. Besides, fsQCA also has the following advantages (Du and Jia, 2017): (1) it is suitable for investigating the causal asymmetry between conditions and results (Rihoux and Ragin, 2009), which can further compare the configurations that lead to the emergence and disappearance of results and broaden its theoretical interpretation dimension of specific research problems; (2) combining the benefits of quantitative and qualitative research and identifying the mechanism of action of condition variables based on a cross-case comparison of large, medium, and small samples (Ragin, 2008; Berg-Schlosser and De Meur, 2009; Crilly et al., 2012; Greckhamer et al., 2013; Greckhamer, 2016), which not only makes up for the lack of external promotion of the original qualitative research, but also breaks the necessary restriction on a large number of samples in quantitative research; and (3) since there are more than one conditional configuration, that causes specific results, which is equivalent (Fiss, 2011), using fsQCA, we can understand the internal driving mechanism that leads to different results in different situations by investigating these equivalent configurations, and discuss the adaptation and substitution relationship between conditions, i.e., “All roads lead to Roma” effect.

### Samples

Select the returning entrepreneurial teams with similar conditions all over the country and carry out a questionnaire survey to collect members’ demographic attributes. To ensure the validity, reliability, and recovery rate of the questionnaire, this study screened the subjects to ensure that they are the core personnel of the entrepreneurial teams and distributed two types of questionnaires to every entrepreneurial team: (1) the questionnaire for members’ information (see Supplementary material), which collected the heterogeneity characteristics of each member of the team in the form of multiple-choice questions, and the distribution quantity depends on the number of team members; (2) the performance measurement table (see Table 1) adopts the Likert 5-point scale to collect data, and takes the weighted average as the score to measure the entrepreneurial performance of every new generation of migrant workers’ returning entrepreneurial team. Each team will only issue one copy, and the team leader will fill in the evaluation sheet according to his own experience and actual business circumstance. Make provisions on the validity of the recovered questionnaire: when each entrepreneurial team participating in the survey submits one performance measurement table and no less than three questionnaires for members’ information, and all the questions in the questionnaire are answered, the questionnaires submitted by the team will be confirmed as valid ones. This study distributed 229 member information collection questionnaires and 40 performance measurement tables. For pre-survey which selects the faultlines for further research, 221 member information collection questionnaires from 45 teams were collected, and the effective rate was about 96.5%. For fsQCA which summarizes the faultline configurations, 149 member information collection questionnaires were collected, and the effective rate was about 65.1%; 32 effective performance measurement tables, with an effective rate of 80%. The descriptive statistics of team member attributes are shown in Table 2.

**TABLE 1:** Performance measurement scale of returning entrepreneurial team.

Indicators	Items	Factor loads		α
Inner indicators (50%)	1. The production capacity of the enterprise is stable	0.84		0.89
	2. Often launches new products or services	0.75		
	3. Product quality makes customers satisfied	0.80		
	4. Employees can work efficiently	0.77		
	5. The enterprise has the confidence to survive and operate	0.71		
Outer indicators (50%)	1. Rapid turnover growth		0.59	0.86
	2. Rapid profit growth		0.67	
	3. The enterprise has sufficient working capital		0.55	
	4. The enterprise has a stable market share		0.69	
	5. The market share position and reputation of the enterprise has gradually increased		0.87	

**TABLE 2:** Descriptive statistics.

Attributes	Measurement items	Sample size	Percentage (%)
*FLS*_*age*_	The post-1980s generation	66	44.3
	The post-1990s generation	47	31.5
	The post-2000s generation	2	1.3
	Others	34	22.8
*FLS*_*gender*_	Male	104	69.8
	Female	45	30.2
*FLS*_*background*_	Urban	58	38.9
	Rural	91	61.1
*FLS*_*expertise*_	Economic management	32	21.5
	Science and technology	54	36.2
	Other types	63	42.3
*FLS*_*risk*_	Adventure	44	29.5
	Intermediate	60	40.3
	Conservative	45	30.2

### Measurement and calibration

#### Antecedent: Team faultline strength

The FLS team faultline strength measurement was proposed by Shaw (2004), which can calculate the team faultline strength of each feature and deal with the calculation of faultline strength between multiple features and subgroups more flexibly than other faultline strength measurements. And the measurement results can comprehensively reflect the homogeneity level within the same subgroup and the heterogeneity level between different subgroups across the faultlines. The strength of the faultlines can better reflect the power comparison and polarization relationship between the subgroups. By collecting the demographic characteristics of the members of the new generation of returning migrant workers’ entrepreneurial teams, this study realizes the FLS measurement to calculate the strength value of the faultlines of each attribute team. The algorithm has strong flexibility and can measure the strength of each demographic faultline, such as age, gender, and so on, so that this research can be carried out based on taking each demographic faultline as an antecedent condition, as shown in the following formula.


F⁢L⁢S=I⁢A×(1-C⁢G⁢A⁢I)

In the above formula, *IA* means the index of alignment in each subgroup, whose value is between 0 and 1; *CGAI* means the cross-group alignment index, which is also between 0 and 1; (1-*CGAI*) indicates the cross-group heterogeneity, which illustrates the diversity among subgroups. The FLS of each team can be found in the Supplementary material.

#### Outcome: Performance of returning entrepreneurial team

As far as the research on the performance of the returning entrepreneurial team is concerned, due to the characteristics of small scale, low income, poor stability, low entrepreneurial satisfaction, and inconvenient disclosure of financial and market data, the objective performance will be limited by poor comparability and inconvenient access. Therefore, this study selects subjective indicators as the basis of entrepreneurial performance measurement. We draw lessons from existing studies and use the Likert 5-point scale to collect performance information such as financial, market, product, employee, and organizational satisfaction (Choi and Thomas, 2010; Zhu and Xie, 2012; Shen et al., 2013) and divide the items into two categories: inner indicators and outer indicators. The weighted average of the sum of the two parts of indicators is used as the observation value of the comprehensive evaluation index, as shown in Table 1 below. The Cronbach’s α of the subjective performance measurement questionnaire reaches 0.923, much higher than the acceptable threshold of 0.7, further suggesting that the internal consistency and good validity of the scale. The following formula explains the calculation process of the entrepreneurial performance.


$$P\,e\,r\,f\,o\,r\,m\,a\,n\,c\,e=0.5\,\sum_{i=1}^{n}i\,n\,n\,e\,r_{i}+0.5\,\sum_{j=1}^{m}o\,u\,t\,e\,r_{j}$$

In the above formula, *inner*_*i*_ represents the *i*-th inner indicator in Table 1, while *outer*_*j*_ represents the *j*-th outer indicator in Table 1; *n* and *m* is the amount of the inner and outer indicators, respectively. The performance score of each team can be found in the Supplementary material.

#### Research framework

Based on the FLS faultline strength measurement and the fsQCA, this study will take the entrepreneurial team performance as the outcome variable and take the demographic faultlines in the entrepreneurial teams of the new generation of returning migrant as the antecedents for configuration analysis according to the dimensions of information-decision type, role-motivation type, and background-experience type of faultlines, exploring the causal relationship between the various faultline configurations and the performance of returning entrepreneurial teams.

**FIGURE 1:**
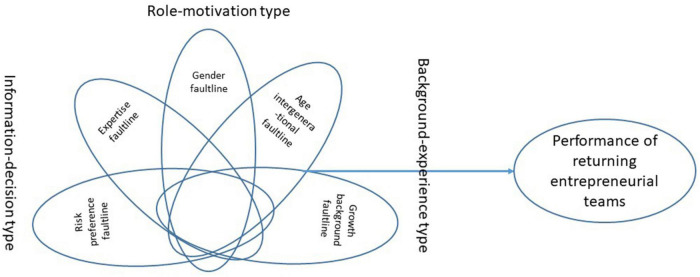
Research framework.

For selecting the most suitable faultlines reflecting the subgroup diversity of the new generation of migrant workers’ entrepreneurial teams, this study carried out a pre-survey conducted the principal component analysis (PCA) and factor analysis on the main team faultlines (see factor loads in Table 3). Three main categories or dimensions of faultline are extracted: information-decision type, role-motivation type and background-experience type, which cover about 68% of the information of original faultlines. To avoid the “limited diversity” in the QCA caused by too many antecedents (Berg-Schlosser and De Meur, 2009; Greckhamer, 2016), this study further screened the existing demographic faultlines, retained the faultlines with a factor load greater than 0.8 in each type as the representatives of antecedents. The final research framework is shown in Figure 1.

**TABLE 3:** Principal component factor coefficient matrix.

Faultline	Categories or dimensions		
	Information-decision	Background-experience	Role-motivation
*FLS*_*risk*_	**0.82**		
*FLS*_*expertise*_	**0.81**		
*FLS*_*edu*_	0.78		
*FLS*_*age*_		**0.93**	
*FLS*_*background*_		**0.81**	
*FLS*_*gender*_			**0.81**
*FLS*_*motivation*_			0.57
*FLS*_*role*_			0.42

## Fuzzy-set qualitative comparative analysis

### Calibration

Before the qualitative comparative analysis, it is required tocalibrate the fuzzy set data at first. A fuzzy set can be regarded asa continuous variable to represent the degree of membership between “Fully in” and “Fully out.” The process of assigning collective membership to these cases is called calibration (Schneider and Wagemann, 2012; Du and Jia, 2017). Given that the antecedents primarily reflect the strength of each faultline within each new generation of migrant workers’ entrepreneurial teams, and the outcome is the total score on the scale, which is the actual measured value, the mechanical anchor is used to calibrate the data set (Ragin, 2008), the “fully in” value is set as the upper quartile, and the “crossover” is set as the median point of the data set, then the “fully out” value is set as the lower quartile of the dataset. The anchor points of each conditions are determined in Table 4.

**TABLE 4:** Calibration anchor points of each variable.

Variable		Anchor point		
Type	Faultline	Fully out	Crossover	Fully in
Information-decision	*FLS*_expertise_	0.0425	0.0800	0.1475
	FLS_risk_	0.0500	0.1150	0.1875
Role-motivation	*FLS*_gender_	0.0525	0.1150	0.2025
Background-experience	FLS_age_	0.0300	0.0900	0.1100
	FLS_background_	0.0000	0.1200	0.2550
Outcome	Team performance	20.500	21.500	22.375

### Necessity analysis

A necessary condition can be regarded as a superset of the result. Rihoux and Ragin (2009) indicated that if the necessary condition is included in the truth table analysis, it may be removed from the solution included in the “logical remainder,” that is, the necessary condition may be eliminated by the parsimonious solution. Therefore, before analyzing the configurations, it is also necessary to check the necessity of each condition separately, then analyze the sufficient conditions that cannot be used as the necessary conditions alone, and screen the configuration with the greatest explanatory power for the target case by using the method of “Boolean algebra minimization.” When analyzing the necessary conditions, attention should be paid to the consistency between each condition and the outcome. If the consistency is greater than 0.9, the condition constitutes the necessary condition for producing the result.

It can be seen from Table 5 that the necessity of the conditions of every single faultline strength affecting the high or non-high entrepreneurial performance of the new generation of returning migrant workers does not exceed 0.9, which does not constitute a necessary condition. It is indicated that the strength of each demographic faultline generally has a weak explanation for the result variable of entrepreneurial team performance of the new generation of migrant workers. Therefore, these antecedents, namely all the demographic faultlines will be included in the fsQCA to explore the configurations leading to high and non-high entrepreneurial performance.

**TABLE 5:** Necessity test results.

		Outcome			
Antecedent		High performance		Non-high performance	
Type	Faultline	Consistency	Coverage	Consistency	Coverage
Information-decision	Strong FLS_risk_	0.5273	0.4994	0.5829	0.6256
	Weak FLS_risk_	0.6047	0.5613	0.5335	0.5613
	Strong FLS_expertise_	0.4327	0.4171	0.6335	0.6922
	Weak FLS_expertise_	0.6807	0.6210	0.4665	0.4824
Role-motivation	Strong FLS_gender_	0.5747	0.5445	0.5082	0.5458
	Weak FLS_gender_	0.5207	0.4830	0.5759	0.6054
Background-experience	Strong FLS_age_	0.6193	0.5752	0.5288	0.5567
	Weak FLS_age_	0.5227	0.4946	0.5964	0.6397
	Strong FLS_background_	0.6293	0.5680	0.5500	0.5626
	Weak FLS_background_	0.5153	0.5026	0.5776	0.6385

### Analysis of faultline configurations

The following analyzes the team faultline configurations that lead to the high and non-high performance of the new generation of migrant workers’ returning entrepreneurial teams. These different configurations represent the combination of different types of team faultlines that achieve the same result (high entrepreneurial performance or non-high entrepreneurial performance). At the same time, the configurations found in this work are named after the process of configuration theory (Furnari et al., 2020).

Firstly, referring to the existing research, this article sets the consistency threshold and PRI consistency threshold to 0.8 and 0.65, respectively, and sets the case frequency threshold to 1 to carry out the configuration analysis of the antecedent conditions. Secondly, the intermediate solution and the parsimonious solution are compared to distinguish the core conditions and the peripheral conditions. The criterion for distinguishing is that when a condition appears in the intermediate solution and the parsimonious solution at the same time, it will be regarded as the core condition; when a condition only appears in the intermediate solution, it will be regarded as a peripheral condition. The analysis results are shown in Table 6. The tagging method of Ragin and Fiss (2008) is followed in the table. The condition variables appear with •, and absent with ⊗; the large circle represents the core condition and the small circle represents the peripheral condition. A blank cell indicates that the conditional variable is irrelevant (present or absent). Among them, four types of faultline configurations affecting the entrepreneurial performance of the new generation of migrant workers’ returning entrepreneurial teams can be obtained, which respectively constitutet he sufficient conditions for the new generation of migrant workers’ high and non-high returning entrepreneurial performance. In addition, the coverage index reflects the proportion of cases that can be explained by the particular configuration and the explanatory power of the configuration to the case outcomes.

**TABLE 6:** Faultline configurations.

		Outcome					
Antecedent		High performance		Non-high performance			
Type	Faultline	H1a	H1b	NH1a	NH1b	NH2	NH3
Information-decision	FLS_risk_	⊗	•	⊗	⊗	🌑	🌑
	FLS_expertise_	⊗	⊗	🌑	🌑	•	🌑
Role-motivation	FLS_gender_	•	⊗	⊗	⊗	🌑	
Background-experience	FLS_age_	🌑	🌑		•		
	FLS_background_	🌑	🌑	⊗		⊗	•
Consistency		0.78	0.80	0.93	0.94	0.89	0.96
Coverage		0.20	0.21	0.17	0.14	0.20	0.20
Unique coverage		0.15	0.16	0.07	0.05	0.13	0.11
Solution consistency		0.78		0.93			
Solution coverage		0.36		0.50			

Generally, the coverage of the overall solution of the configurations producing high entrepreneurial performance has reached about 0.39, indicating that about 39% of the cases of high entrepreneurial performance of the new generation of returning migrant workers can be explained by this type of configuration (H1a or H1b); the coverage of the overall solution of the configuration generating non-high entrepreneurial performance reached about 0.53, indicating that about 53% of the cases of non-high entrepreneurial performance of the new generation of returning migrant workers can be explained by these three types of configurations (NH1a or NH1b; NH2; and NH3). The coverage indicators in Table 5 show that all configurations have strong persuasion in explaining the high or non-high performance of the new generation of migrant workers returning to their hometowns to start businesses.

### Robustness test

To ensure the reliability and validity of the configuration model, the robustness of the configuration analysis results was tested. The result shows that the four configuration types in the testing model are generally consistent with the prominent characteristics of each configuration in the original model, indicating that the research conclusion is relatively stable.

## Results

### Configuration types of faultlines producing high entrepreneurial performance

#### Type H1: The background-experience actuation type

It is characterized by the appearance of the strong background-experience type of faultlines and weak information-decision type of faultlines as the core conditions. It contains two subtype configurations, with the emergence of gender faultline and risk preference faultline as the peripheral conditions, respectively. On the one hand, although the members of the entrepreneurial team of the new generation of returning migrant workers have different social attributes, under the influence of the common identity of the “new generation of migrant workers,” entrepreneurial members can sensitively confirm their common identification in their social role and perceive their sense of belonging to the group (Li and Wang, 2014), which is an important reason why background-experience type of faultlines rarely plays a positive role rather than a negative role in other management scenarios. The common background and experience of the “new generation of migrant workers” make them highly united in the identity homogeneous entrepreneurial team and make the team more cohesive, which is enough to resolve the conflict caused by the different mindsets shaped by the specific growth environments and life experiences (Bezurukova et al., 2009). Based on this, with the help of the diversity of experience and mindsets mentioned before, returning entrepreneurs can cause a situation in which the old leads the young or the strong leads the weak, improving the overall entrepreneurial ability of the team. That is what we call “the guidance mechanism.”

On the other hand, the weak information-decision faultlines of the team are not caused by the lack of knowledge heterogeneity of subgroups but by different professional knowledge held by each member. Taking team 8160 (see Supplementary material) as an example, the expertise of four members covers all three types involved in the research, i.e., the diversity of professional knowledge at the member level is too strong, resulting in the “medium” heterogeneity in the subgroup level that can contribute to the occurrence of strong faultlines (Lau and Murnighan, 1998). Therefore, while maintaining the diversity of information-decision, teams with the H1 configuration type can minimize the confrontational subgroup conflicts and promote the generation of high returning entrepreneurial performance.

In addition, risk preference (H1a) or gender faultline (H1B) can replace each other as a peripheral condition, that is, the occurrence of any and only one of them can affect judgment and cognition during the decision-making process. Driven orderly by the guidance mechanism, through the balance of power at the subgroup level, it plays a stabilizing role in decision-making, suppressing the tendency of blind and perceptual decision-making, then contributing to high entrepreneurial performance. That is what we define as “the balance mechanism.”

### Configuration types of faultlines producing non-high entrepreneurial performance

#### Type NH1: The guidance-balance lacking type

It is characterized by weak risk preference faultline, weak gender faultline, and strong information-decision type of faultlines as the core conditions, including two subtypes. The returning entrepreneurial team under this type of configurations lacks not only the long-term experience guidance brought by the diversity of growth background, but also the balance and stability for decision-making. In the absence of guidance and balance mechanism, team members will form highly similar deep-level cognition based on the alignment of growth background, such as risk preference, which will lead to a high degree of unity in their views on decision-making, and there are few objecting voices in the decision-making process. Nevertheless, due to the extreme disparity of subgroup size (such as 1:n), weak subgroups will be outnumbered by other strong ones and it will be difficult to have a significant impact on the team’s decision-making in order to reduce the frequency of opinion expression and weaken the balance in the later development of the team (Lau and Murnighan, 1998).

Taking team 9227 (see Supplementary material) as an example, most of its members are highly similar in gender and growth background (male; rural), and their risk preference all tends to be “intermediate.” Due to the convergence of cognition, the team will be easy to reach an agreement with the decision-maker based on one-sided experience and be trapped in the dilemma of using abundant, diverse information resources to implement irrational entrepreneurial behavior (such as investing in high-risk or low return projects). Under this circumstance, the positive impact of information-decision heterogeneity cannot offset the negative impact of irrational decision-making judgment, resulting in non-high performance.

#### Type NH2: The role-cognition conflict type

Its prominent feature is that the strong gender faultline and strong risk preference faultline are the core conditions, and the growth background faultline is missing. In this configuration, gender roles and their associated risk preference differences exist at the same time. It is generally believed that women are good at emotional decision-making while men are good at rational decision-making; risk preference directly affects risk choice. A considerable part of the entrepreneurial groups of the new generation of returning migrant workers is female. They can have male characters, such as risk-taking or good leadership skills, as well as feminine characteristics, such as sensitivity and good interpersonal communication (Zhou and Cui, 2021). Influenced by traditions and stereotypes in gender roles, male entrepreneurs in the new generation of migrant workers may have potential conflict points with female entrepreneurs with masculine cognitive characteristics. In addition, lacking the driving mechanism in the background-experience actuation type, the social experience of each member is so similar that the status of members with the same growth background is more equal since there is no authoritative subgroup composed of older or experienced members in the team. Different from the situation in which either of the two occurs alone, when the strong gender faultline and the strong risk preference faultline occur at the same time, due to the integration of identity and cognition discrepancy, it is easier to cause fierce conflicts between subgroups in the process of decision-making balance, reducing team cohesion and produce negative effects.

Taking the typical team 8744 (see Supplementary material) as an example, the team members are highly resembled in the growth background, i.e., “post-80s; rural,” and there is no subgroup with long-term experience advantage as the authoritative guides to instruct the team’s decision-making; the gender subgroups and the risk preference subgroups have observable overlap. In the case of poor guidance, it is very likely to produce conflict in risk decision-making between subgroups to reduce decision-making efficiency and team cohesion and negatively affect team performance.

#### Type NH3: The information-decision polarization type

Its prominent feature is that two high-intensity information-decision faultlines appear as the core conditions at the same time. The emergence of this type of faultline configuration verifies the theory of Georgakakis et al. (2017) and Lau and Murnighan (1998) once again: even the faultline of information-decision team, which is generally considered to have a positive impact, can lead to a highly internal division and task conflict in the team by dividing the whole team into subgroups with equal scale, resulting in low team performance. Specifically, the information difference between subgroups is different from that among subgroup members: as information subgroups with equal power, the professional views of members in the same subgroup are based on the common knowledge background on the decision making process, which is easy to gain the acknowledgment and support from other members within subgroup; driven by small collective groups, individual differences within the team will rise to differences between subgroups; in order to obtain stronger support within the subgroup, members in subgroup tend to produce “polarization” in opinions (Lau and Murnighan, 1998), which will significantly deepen the conflict of dissents between the subgroups, thus strengthening the “group-in and group-out” effect, limiting information sharing and seriously weakening the actual utilization of diversified resource pools, causing extremely adverse impact on team performance finally (Georgakakis et al., 2017).

Taking team 3314 (see Supplementary material) as an example, the strength of its expertise faultline is strong, i.e., “Other types vs. Economic management,” and the size of the expertise subgroups is similar (3:2). Besides, it is highly consistent with the risk preference faultline (Adventure vs. Intermediate). The observable strong information-decision faultlines, which can predict the extent of its task conflict and the polarization of the subgroup, make the team rank in the penultimate in entrepreneurial performance among the 32 teams.

## Conclusion

Based on the team faultline theory and using a qualitative fuzzy set comparison analysis, aiming at the research issue of “how do the different types of faultline configurations affect the entrepreneurial team performance of the new generation of returning migrant workers,” this article analyzes the different combinations among the information-decision type, the role-motivation type and the background-experience type of faultlines in 32 new generation of returning migrant workers’ entrepreneurial teams with similar conditions in China.

Through analysis, we obtain four types of faultline configurations: background-experience actuation (H1); guidance-balance lacking (NH1); role-cognition conflict (NH2); information-decision polarization (NH3). Based on the results we gained, this study draws the following conclusions: (1) the team faultline configuration driven by background-experience differences has a positive impact on the performance of the new generation of migrant workers’ returning entrepreneurial team through the formation of identity, driving and guidance, and balance mechanism in the team; (2) the guidance-balance lacking type, role-cognitive conflict type and information-decision polarization type of faultline configurations negatively affect the performance of returning entrepreneurial teams through irrational decision-making and polarization conflict between drama teams; (3) there is causal asymmetry between the conditions of team faultlines and the results of team performance. For instance, the existence of a strong gender faultline and strong risk preference faultline may lead to non-high performance (NH2), but non-high performance does not necessarily mean that there are strong gender faultline and strong risk preference faultline (NH1) in the team; and (4) there is an imbalance in the number of types of team failure configurations that produce high-performing and non-high-performing the returning entrepreneurial teams of the new generation of migrant workers.

The configuration types that produce non-high performance are significantly more than those that produce high performance, indicating that the urgency to avoid weaknesses is greater than to develop strengths. The problem of team faultlines in the new generation of migrant workers’ returning entrepreneurial teams urgently needs to be properly solved under the guidance of scientific management theory.

## Discussion

### Theoretical contributions

The contribution of this study to the development of theory mainly lies in three aspects: research methods innovation, local managerial scenarios, and practical instructions. Among them, the innovations in methods and scenarios contribute to the development of the faultline theory directly, while the instructive suggestions contribute to the scientific management of the human resource management and the increase of the business performance of the new generation of migrant workers’ entrepreneurial teams, helping them to successfully run their businesses in their rural hometown.

In terms of the research method, we have used the fsQCA method to study the effectiveness of team faultlines on team performance and select the information-decision type, the role-motivation type, and the background-experience type of faultlines discovered in the returning entrepreneurial teams of the new generation of Chinese migrant workers as the dimensions of antecedent conditions. To a certain extent, this analysis of each specific faultline configuration as a whole corresponds to the prospect of some scholars at present exploring the interaction of different types of faultlines at present, and provides empirical confirmation. It will help give a specific reference for interpreting the differences in the effectiveness of the faultlines on team results and better understanding the mechanism of influence of the team faultlines. In a way, it can explain that the instability of the negative or positive effects of the simple social-category faultline or information-related faultline originates from the different configurations of various faultlines types and is limited to the guidance and balance mechanism, polarization effect and other factors caused by different configurations, which is a one-sided representation of the overall effect of the configurations.

In terms of the research scenario, we have taken the new generation of migrant workers in China as the special focus, aiming at enriching the management theory of the new generation of migrant workers returning to their hometowns to start businesses. The previous research on the application of the team faultline theory exists mainly in the research field of the top management team of publicly traded companies, e.g., the CEO-TMT interaction, etc., which lacks attention to small and medium-sized entrepreneurs and entrepreneurial teams. Small and medium-sized entrepreneurial teams, especially those resembling the entrepreneurial teams of the new generation of migrant workers, are characterized by a lack of management skills and experience, significant vulnerability, and a survival purpose greater than the development purpose in operational and managerial activities due to their uniqueness characteristics such as resource scarcity and poor team configuration. It has clear differences from the management context of large listed companies, which is a research topic that can be extended by the theory of team faultline at present. On the one hand, we can find the rationality of the existing team faultline theory and the special differences between various management subjects; on the other hand, it adds a new understanding to the related theories about the entrepreneurship of the new generation of returning migrant workers and provides theoretical support for putting forward the specific strategies of entrepreneurship management of this group.

Last but not least, In terms of the practical instruction, aiming at optimizing the diversity structure and taking advantages of the heterogeneous resources, we eventually gain four types of faultline configurations in the new generation of migrant workers’ entrepreneurial teams, acting as a basis of making the countermeasures to maximize the information-decision advantages of the faultlines caused by various dimensions of expertise and risk control, and minimized the “group-in” and “group-out” cognitive conflicts caused by the psychological factors including roles, motivations, and cognitive backgrounds. Specifically, we have proposed several concepts like “the guidance mechanism” and “the balance mechanism” to explain why the migrant worker entrepreneurs need an experienced leader/leaders who can utilize his/her/their rich expertise and persuasive authority to guide and instruct other members, as well as balancing the power among subgroups to mediate the potential subgroup polarization and cross-group quarrels which damage the consolidation of the team. Then we have also advocated to watch out the cognitive conflicts based on the difference in gender roles, encouraging team members of different gender roles to respect the equal rights of expressing opinions and making decisions in the entrepreneurial practice, so that more constructive views can help to make reasonable, comprehensive decision in business. From an international perspective, conclusions and implications of our study can also be utilized by migrant worker entrepreneurs planning to start their businesses under similar conditions of member heterogeneity in Southeast Asia, Latin America, Africa, and other developing areas.

### Managerial implications

Based on the four types of faultline configurations, the following specific implications are put forward for the management of the returning entrepreneurial team of the new generation of migrant workers in both China and other similar developing areas, where the rapid industrialization shapes the migrant workers’ heterogeneous social backgrounds, knowledge and skills, values, and cognitions:

First, the entrepreneurs of the new generation of returning migrant workers should take advantage of the background-experience differences of team members and design appropriate guidance and balance mechanisms. Specifically, returning entrepreneurs ought to build the suitable background-experience type of faultlines in the entrepreneurial team, paying attention to the personnel configuration based on the differentiation of growth background and social experience, as well as giving play to the leading and guiding role of the authoritative subgroups through the application of long-term cognitive reserve to form a cooperation situation in the whole entrepreneurial team where the old ones lead the young ones and the strong ones lead the weak ones, leading to the improvement of the entrepreneurial ability of the team; based on this, make use of the differences in gender preference and risk cognition at the subgroup level to set up a reasonable and effective decision-making balancing mechanism to promote the team’s rational decision-making.

In the practice of returning to their hometowns to start a business, as Chinese rural society is deeply influenced by the traditional “society of human relationship” and stereotypes about gender roles, returning entrepreneurs usually choose their hometowns relatives or friends who live together for a long time to form the entrepreneurial team and the members are mostly men. As a result, the entrepreneurial teams of the new generation of migrant workers often have a similar growth background and single-gender composition, lacking the effective “guidance” and “balance” mechanism. In order to change this situation, the new generation of migrant worker entrepreneurs should actively identify and diversify the growth experiences of their relatives and friends in the preparation of the team. For example, they can introduce their elderly male cousins and their town fellow workers with long-term and rich learning and working experience in the cities into the entrepreneurial team, and cooperate with their sisters who have been familiar with rural production and living conditions since a young age. Considering the urban demand, the team can better adapt to rural production, coordinating supply and demand to make rational marketing decisions and accurate operations in production. In addition, local governments should continue to use economic subsidies and social welfare to encourage migrant workers to return to their hometowns and set up businesses, and focus on introducing the returning elites to the local entrepreneurial teams, and build “the guidance mechanism” in the teams accurately.

Second, returning entrepreneurs should guard against one-sided “efficient” decision-making caused by cognitive convergence. As mentioned above, the lack of guidance and balancing mechanisms will lead to the convergence of decision-making within the entrepreneurial team and the absence of valuable objections, resulting in the illusion of “high efficiency” of entrepreneurial decision-making. The new generation of migrant workers usually maintains a simple democratic decision-making concept of “the minority obeys the majority” in the practice of management. However, there is also a sort of neglect of in-depth attention and analysis of minority opinions. As a management cognitive problem within the entrepreneurial team, the returning entrepreneurial teams should pay attention to the process control of decision-making, especially the communication and control during and after decision-making. Specifically, when making a decision, the decision-maker needs to get rid of the myth of “all agree at one time,” avoiding the preset tendency as much as possible, then adopting brainstorming and other methods to emerge all sorts of views, paying attention to the reasonable points of the dissents of minority, so as to prevent from collectively ignoring important issues; after making the decisions, returning entrepreneurs ought to reinforce the monitoring of the decision-making results, such as paying close attention to the changes of quantitative performance such as sales, profits and market share, revealing problems in time and giving feedback, correcting the deviation of irrational decision-making, and preventing the continuous waste of resources. Outside the team, the government should guide all sectors of society strengthening business decision-making by using modern digital technologies such as big data or artificial intelligence to build platforms to support business decision-making, and provide entrepreneurial cases and other information services to enhance the cognitive diversity of the new generation of returning entrepreneurs and to break through the “cognitive cocoon” of decision-making.

Third, coordinate the role-cognition conflict, especially the cognitive differences and conflicts based on gender decision-making. In teams with faultline configuration of role-cognition conflict, due to the absence of authoritative members or subgroups and the high correlation between gender decision-making subgroups and risk preference subgroups, the subgroups with multiple alignments inside are equally sized, and the role conflict is particularly intense. In China’s rural context, the stereotype of gender roles will exacerbate the cognitive tear of entrepreneurial teams. On the one hand, the duality and epochal characteristics of the new generation of female migrant workers allow the entrepreneurs to recognize their special advantages in the embedding of industrial networks and to translate them into high entrepreneurial willingness and entrepreneurial action; on the other hand, it reflects that the male returning entrepreneurs still need to get rid of the potential influence of traditional gender notion. Based on this, the male members of the entrepreneurial teams of the new generation of returning migrant workers should change the traditional stereotypes, be open-minded, respect the status and role of female members, and face up to their contribution to the embedding of industrial networks; female entrepreneurial members should make full use of their advantages in the aspect of interpersonal communication, actively coordinating the relations among team members as well as maintaining team cohesion while actively coming up with their unique opinions. At the same time, when there are difficult role-cognition conflicts within the entrepreneurial teams, members can also seek the assistance of external forces of the team and take the introduction of authoritative members as a breakthrough to build a guidance and balance mechanism. For example, governance organizations such as the village committees can take this opportunity to dispatch entrepreneurial assistants or consultants to accurately connect to the problematic teams in order to coordinate role conflicts from outside to inside or from top to bottom and improve the internal relationship of the team.

Fourth, attention should be drawn to the management of information-decision faultlines. From a management practice perspective, the most viable strategy should be to manage the information-related faultlines well, rather than simply avoiding them. Therefore, the task of the new generation of returning migrant workers entrepreneurs is to control the information-decision conflict of subgroups and maximize the resource advantages of information differences: for one thing, the new generation of migrant workers’ entrepreneurial teams need to reduce the information-decision differences at the subgroup level, resolving the heterogeneity of knowledge and information to the level of team members, and forming strong diversity under the condition of weak faultlines. This will significantly reduce the polarization and conflict of rival subgroups of a similar size and maintain the diversity of information resources of the team. Therefore, for the staffing of returning entrepreneurial teams, team leaders need to focus on building their teams with unique expertise fields for each member rather than with subgroups of multiple professional fields. For example, a four-person team can have one member who has professional knowledge of management, one member who has manufacturing technology, one member who has network technology knowledge, and one member who has communication and bargaining skills, rather than being composed of two management professionals and two production and manufacturing personnel. For another thing, they should use various “bridges” to communicate with each information-decision subgroup, emphasizing the cross-border ability of the members and the role of the “new generation migrant workers” identity in breaking the “group-in and group-out” effect and improving the overall consolidation of an entrepreneurial team to form integration at the level higher than the subgroups, and curb the information fragmentation and conflict in the information-decision faultline, enhancing members’ willingness to cooperate across subgroups and share the overall risks of the team.

In addition, it should be emphasized here that the problem caused by the information-decision faultlines in the entrepreneurial team of the new generation of migrant workers is essentially the limitation of the choice of professional directions of the new generation of migrant workers when receiving advanced or vocational education, while the root of this limitation lies in the lack of basic education resources received by the new generation of migrant workers and the low ability to absorb knowledge, resulting in their lack of qualification for enrolling in further education. In order to improve the information diversity of the next generation of returning entrepreneurs, it is important to highlight the progress made in the balanced development of urban and rural education and the improvement of the basic education level of the children of the new generation of migrant workers based on further support of the employment and entrepreneurship training policies of the new generation of migrant workers. It is also crucial to increase the targeted enrollment of children of migrant workers in urban public schools and improve the quality of rural teachers, as well as build online educational platforms to share the quality educational resources in both urban and rural areas; migrant workers should be encouraged to involve in all kinds of studies, especially business management, computer science and technology, or other special fields related to rural entrepreneurship to develop more entrepreneurial experts in the new generation of the returning migrant workers to adapt to this digital and knowledge economy age, as well as the strategy of rural revitalization.

### Limitations and future works

This article examines and analyzes the impact of team faultline configurations on the entrepreneurial performance of the new generation of migrant workers returning home using the method of qualitative comparative research with fsQCA. Due to the subjective and objective constraints brought by the competence of the researchers, the applicability of methods, and the research object, there is still great room for progress and development in relevant theoretical and empirical research:

Firstly, because of the limited availability of case data, this study only carries out QCA analysis on 32 new generation migrant workers’ returning entrepreneurial teams, which affects the external promotion of the empirical results to a certain extent. There are problems of limited diversity worthy of attention as well. Future research can consider expanding the capacity of the cases and further verifying the relevant theoretical research results.

Secondly, this study only focuses on the formation and influence of the faultlines configurations in the new generation migrant workers’ returning entrepreneurial teams under the static condition. It also lacks the consideration of the dynamic development of the entrepreneurial teams and the quantitative research on measuring the contribution of various antecedents in the configuration. With the advent of dynamic QCA and NCA research, future research will focus on the use of time-series comparative analysis, qualitative comparative analysis over multiple periods, comparative analysis of necessary conditions, and other methods, combined with Lau and Murnighan’s theoretical conception of faultline evolution under the dynamic development of the team, to make a more in-depth exploration on the relationship between the trajectory of the change of faultline configurations and that of the change of entrepreneurial performance in the returning entrepreneurial team of the new generation of migrant workers, as well as the fine-grained analysis of the interaction between various types of faultline configurations and the performance.

In addition, this study chooses the returning entrepreneurial team of the new generation of migrant workers as research object, limiting the theoretical scope for the explanation. Future research will examine whether the conclusions of this study can be extrapolated to other management issues, such as top management teams of listed companies, entrepreneurial teams of college students, etc., which will enhance and practice the universal value of relevant conclusions for management theory.

Finally, in terms of variable selection, the focus of variable selection will gradually change as relevant research develops, moving up from faultlines based on demographic characteristics to faultlines based on complex and abstract criteria such as social niche, cognition, and social capital, which is helpful for researchers and practitioners to understand the subtle role of team heterogeneity on management behavior and results under the complex relationship between human nature and society.

## Data availability statement

The original contributions presented in this study are included in the article/Supplementary material, further inquiries can be directed to the corresponding author.

## Ethics statement

Ethical review and approval was not required for the study on human participants in accordance with the local legislation and institutional requirements. Written informed consent from the patients/participants OR patients/participants legal guardian/next of kin was not required to participate in this study in accordance with the national legislation and the institutional requirements.

## Author contributions

ZQ contributed to the research designing, data collection, analysis, and writing—original draft. KS contributed to the questionnaire designing, data collection, and reviewing the translating version. NZ contributed to the translation and edited the final draft. TS contributed to the methodology and research guidance, approved the final draft. All authors contributed to the article and approved the submitted version.
