# Supplementary material for: Faultline configurations affecting the entrepreneurial team performance of new generation of returning migrant workers in China: An empirical study based on fuzzy-set qualitative comparative analysis
Source: Front Psychol. 2022 Oct 12;13:918128. doi: 10.3389/fpsyg.2022.918128 (PMC9597618; doi:10.3389/fpsyg.2022.918128)
Supplement: Supplementary file 1 [file Table_1.DOCX]

Supplementary Material

# Heterogeneity Attributes under Various Types of Faultlines (The Questionnaire for Members’ Information)

| Background-experience type | Age intergenerational faultline  (${FLS}_{age}$) | A. Post-80s  B. Post-90s  C. Post-00s  D. Others, such as entrepreneurial team participants of the older generation |
| --- | --- | --- |
|  | Growth environment faultline  (${FLS}_{background}$) | A. Urban  B. Rural |
| Information-decision type | Educational level faultline  (${FLS}_{edu}$) | A. Primary school and below  B. Junior high school  C. Senior high school  D. Technical secondary school or Junior college  E. Bachelor’s degree or above |
|  | Expertise faultline  (${FLS}_{expertise}$) | A. Economic management  B. Science and technology  C. Other types |
|  | Risk preference faultline  (${FLS}_{risk}$) | A. Adventurous  B. Intermediate  C. Conservative |
| Role-motivation type | Gender role faultline  (${FLS}_{gender}$) | A. Male  B. Female |
|  | Entrepreneurial role faultline  (${FLS}_{role}$) | A. Inventor  B. Founder  C. Developer |
|  | Entrepreneurial motivation faultline  (${FLS}_{motivation}$) | A. Economic-oriented  B. Social-oriented  C. Achievement-oriented |

# Results of the Measurement

| **team_id** | **ageFLS** | **genderFLS** | **backgroundFLS** | **professionFLS** | **riskFLS** | **score** |
| --- | --- | --- | --- | --- | --- | --- |
| 1064 | 0.11 | 0.21 | 0.27 | 0.18 | 0.18 | 22.8 |
| 1333 | 0.16 | 0.07 | 0.31 | 0.21 | 0.09 | 20.8 |
| 1677 | 0.1 | 0.28 | 0 | 0.13 | 0.2 | 20.6 |
| 1876 | 0.15 | 0 | 0.08 | 0.1 | 0.18 | 22.4 |
| 1987 | 0.07 | 0.23 | 0.12 | 0.08 | 0.05 | 22.2 |
| 2854 | 0.03 | 0.05 | 0.05 | 0.1 | 0.1 | 21.2 |
| 2980 | 0 | 0.16 | 0 | 0.04 | 0.1 | 23 |
| 3300 | 0.02 | 0.05 | 0 | 0.08 | 0.08 | 21.4 |
| 3314 | 0.02 | 0.04 | 0.47 | 0.31 | 0.35 | 9 |
| 4159 | 0.05 | 0.15 | 0.24 | 0.14 | 0.23 | 20.4 |
| 4231 | 0.09 | 0.1 | 0 | 0.05 | 0.12 | 19.4 |
| 4440 | 0.06 | 0.18 | 0.18 | 0.07 | 0.08 | 21.6 |
| 4817 | 0.1 | 0 | 0.12 | 0.04 | 0.19 | 21 |
| 4983 | 0.11 | 0.12 | 0.12 | 0.15 | 0.21 | 6.8 |
| 5710 | 0.11 | 0.22 | 0.22 | 0 | 0 | 22.2 |
| 5943 | 0.09 | 0.08 | 0.1 | 0.05 | 0.12 | 21.6 |
| 6604 | 0.27 | 0.22 | 0.31 | 0.03 | 0.12 | 20.4 |
| 6729 | 0.02 | 0.17 | 0 | 0.15 | 0.11 | 22.4 |
| 7235 | 0.03 | 0.06 | 0 | 0.04 | 0 | 21.8 |
| 7440 | 0.03 | 0.06 | 0 | 0 | 0.04 | 21.8 |
| 8160 | 0.15 | 0.11 | 0.3 | 0.06 | 0.2 | 22.8 |
| 8483 | 0.02 | 0 | 0.09 | 0.05 | 0.15 | 23.8 |
| 8530 | 0.1 | 0.05 | 0.26 | 0.11 | 0.17 | 21.4 |
| 8583 | 0.14 | 0.18 | 0.31 | 0.19 | 0.03 | 22.2 |
| 8739 | 0.06 | 0.12 | 0.05 | 0.1 | 0.05 | 20 |
| 8744 | 0.04 | 0.32 | 0 | 0.23 | 0.25 | 12.2 |
| 8885 | 0.12 | 0.23 | 0.23 | 0.03 | 0 | 22 |
| 9081 | 0.09 | 0.08 | 0.19 | 0.05 | 0.12 | 21.4 |
| 9109 | 0.22 | 0.01 | 0.34 | 0.05 | 0.3 | 22.6 |
| 9227 | 0.11 | 0.11 | 0.11 | 0.12 | 0.01 | 20.6 |
| 9326 | 0.01 | 0.31 | 0.16 | 0.15 | 0.1 | 21 |
| 9675 | 0.07 | 0.14 | 0 | 0.04 | 0 | 21.6 |

# Dataset of the Original Information of the Members in the Sample Teams

| **team_id** | **age** | **gender** | **background** | **profession** | **risk** |
| --- | --- | --- | --- | --- | --- |
| 1064 | Post-90s | Male | Rural | Other types | Intermediate |
| 1064 | Others | Male | Rural | Other types | Intermediate |
| 1064 | Others | Female | Rural | Other types | Intermediate |
| 1064 | Post-80s | Female | Urban | Science and technology | Adventurous |
| 1333 | Post-90s | Male | Rural | Economic management | Intermediate |
| 1333 | Others | Male | Urban | Other types | Intermediate |
| 1333 | Post-90s | Female | Rural | Science and technology | Conservative |
| 1333 | Others | Male | Urban | Other types | Adventurous |
| 1333 | Others | Male | Urban | Science and technology | Conservative |
| 1677 | Others | Female | Rural | Economic management | Intermediate |
| 1677 | Post-90s | Male | Rural | Other types | Conservative |
| 1677 | Post-90s | Male | Rural | Economic management | Intermediate |
| 1677 | Others | Female | Rural | Other types | Intermediate |
| 1677 | Post-80s | Male | Rural | Other types | Conservative |
| 1677 | Others | Male | Rural | Science and technology | Adventurous |
| 1876 | Post-90s | Male | Rural | Other types | Conservative |
| 1876 | Post-80s | Male | Rural | Science and technology | Adventurous |
| 1876 | Post-80s | Male | Rural | Economic management | Adventurous |
| 1876 | Post-80s | Male | Urban | Other types | Adventurous |
| 1876 | Post-90s | Male | Rural | Other types | Intermediate |
| 1876 | Post-90s | Male | Rural | Science and technology | Intermediate |
| 1987 | Post-90s | Male | Urban | Other types | Intermediate |
| 1987 | Others | Male | Rural | Science and technology | Intermediate |
| 1987 | Post-90s | Female | Rural | Science and technology | Adventurous |
| 1987 | Others | Female | Rural | Science and technology | Conservative |
| 2854 | Post-90s | Male | Rural | Economic management | Conservative |
| 2854 | Post-80s | Female | Rural | Other types | Adventurous |
| 2854 | Post-80s | Male | Urban | Science and technology | Intermediate |
| 2854 | Post-80s | Male | Rural | Other types | Intermediate |
| 2980 | Post-90s | Male | Urban | Science and technology | Conservative |
| 2980 | Post-00s | Male | Urban | Other types | Conservative |
| 2980 | Post-80s | Female | Urban | Science and technology | Intermediate |
| 3300 | Post-80s | Male | Urban | Economic management | Adventurous |
| 3300 | Post-90s | Male | Urban | Other types | Intermediate |
| 3300 | Post-80s | Female | Urban | Other types | Intermediate |
| 3314 | Post-80s | Male | Urban | Other types | Adventurous |
| 3314 | Post-80s | Female | Urban | Other types | Adventurous |
| 3314 | Others | Male | Urban | Other types | Conservative |
| 3314 | Post-80s | Male | Rural | Economic management | Intermediate |
| 3314 | Post-80s | Male | Rural | Economic management | Intermediate |
| 4159 | Post-80s | Female | Rural | Science and technology | Conservative |
| 4159 | Others | Male | Rural | Economic management | Intermediate |
| 4159 | Others | Male | Urban | Other types | Adventurous |
| 4159 | Post-80s | Male | Rural | Science and technology | Conservative |
| 4159 | Others | Female | Urban | Science and technology | Adventurous |
| 4159 | Others | Female | Rural | Science and technology | Intermediate |
| 4159 | Post-80s | Male | Rural | Science and technology | Intermediate |
| 4159 | Post-80s | Male | Rural | Other types | Intermediate |
| 4159 | Post-80s | Male | Rural | Other types | Intermediate |
| 4159 | Post-80s | Female | Rural | Economic management | Adventurous |
| 4231 | Post-90s | Male | Rural | Economic management | Adventurous |
| 4231 | Post-80s | Male | Urban | Economic management | Intermediate |
| 4231 | Post-80s | Male | Rural | Science and technology | Intermediate |
| 4231 | Others | Female | Rural | Other types | Conservative |
| 4440 | Post-90s | Female | Rural | Science and technology | Adventurous |
| 4440 | Others | Male | Urban | Other types | Intermediate |
| 4440 | Post-90s | Female | Rural | Other types | Intermediate |
| 4440 | Post-90s | Male | Rural | Other types | Intermediate |
| 4440 | Others | Female | Rural | Other types | Conservative |
| 4440 | Post-90s | Male | Rural | Economic management | Adventurous |
| 4440 | Post-80s | Male | Urban | Economic management | Intermediate |
| 4440 | Post-90s | Male | Urban | Other types | Adventurous |
| 4817 | Others | Male | Rural | Other types | Conservative |
| 4817 | Post-90s | Male | Urban | Science and technology | Intermediate |
| 4817 | Post-80s | Male | Rural | Economic management | Intermediate |
| 4817 | Others | Male | Rural | Science and technology | Conservative |
| 4983 | Others | Male | Urban | Other types | Intermediate |
| 4983 | Post-80s | Male | Rural | Science and technology | Conservative |
| 4983 | Post-90s | Female | Rural | Economic management | Adventurous |
| 4983 | Post-80s | Male | Urban | Science and technology | Conservative |
| 4983 | Post-80s | Male | Rural | Science and technology | Intermediate |
| 5710 | Others | Female | Rural | Science and technology | Intermediate |
| 5710 | Post-80s | Male | Urban | Other types | Conservative |
| 5710 | Others | Female | Rural | Other types | Adventurous |
| 5943 | Post-80s | Male | Rural | Science and technology | Intermediate |
| 5943 | Post-90s | Male | Urban | Economic management | Conservative |
| 5943 | Post-80s | Female | Rural | Other types | Intermediate |
| 5943 | Post-90s | Female | Rural | Science and technology | Adventurous |
| 6604 | Post-80s | Male | Rural | Other types | Conservative |
| 6604 | Post-90s | Male | Rural | Science and technology | Adventurous |
| 6604 | Post-90s | Male | Rural | Other types | Adventurous |
| 6604 | Others | Female | Urban | Other types | Intermediate |
| 6604 | Post-90s | Male | Rural | Science and technology | Conservative |
| 6604 | Post-80s | Female | Rural | Science and technology | Conservative |
| 6604 | Others | Female | Urban | Science and technology | Adventurous |
| 6729 | Others | Male | Urban | Economic management | Conservative |
| 6729 | Post-80s | Male | Urban | Other types | Conservative |
| 6729 | Post-90s | Male | Urban | Economic management | Conservative |
| 6729 | Others | Female | Urban | Other types | Adventurous |
| 7235 | Post-80s | Male | Rural | Other types | Intermediate |
| 7235 | Post-90s | Male | Rural | Science and technology | Adventurous |
| 7235 | Post-80s | Female | Rural | Science and technology | Conservative |
| 7440 | Post-90s | Male | Urban | Economic management | Intermediate |
| 7440 | Post-80s | Female | Urban | Other types | Intermediate |
| 7440 | Post-80s | Male | Urban | Science and technology | Conservative |
| 8160 | Post-80s | Male | Urban | Economic management | Conservative |
| 8160 | Post-80s | Male | Urban | Other types | Conservative |
| 8160 | Post-90s | Female | Rural | Science and technology | Intermediate |
| 8160 | Others | Male | Rural | Other types | Adventurous |
| 8483 | Post-90s | Male | Rural | Other types | Conservative |
| 8483 | Post-90s | Male | Urban | Science and technology | Adventurous |
| 8483 | Post-80s | Male | Urban | Other types | Conservative |
| 8483 | Others | Male | Urban | Other types | Adventurous |
| 8530 | Post-80s | Male | Urban | Economic management | Intermediate |
| 8530 | Others | Male | Urban | Science and technology | Conservative |
| 8530 | Post-80s | Female | Urban | Science and technology | Conservative |
| 8530 | Others | Male | Urban | Other types | Adventurous |
| 8530 | Post-80s | Male | Rural | Science and technology | Intermediate |
| 8530 | Post-80s | Male | Urban | Other types | Intermediate |
| 8530 | Post-80s | Male | Rural | Science and technology | Intermediate |
| 8583 | Post-90s | Male | Urban | Other types | Adventurous |
| 8583 | Post-80s | Female | Urban | Other types | Intermediate |
| 8583 | Post-80s | Male | Rural | Science and technology | Intermediate |
| 8583 | Post-90s | Male | Urban | Economic management | Intermediate |
| 8583 | Post-80s | Female | Rural | Science and technology | Intermediate |
| 8739 | Post-90s | Male | Rural | Other types | Conservative |
| 8739 | Post-90s | Male | Urban | Other types | Intermediate |
| 8739 | Post-80s | Female | Rural | Science and technology | Conservative |
| 8739 | Post-90s | Male | Rural | Economic management | Adventurous |
| 8744 | Post-80s | Male | Rural | Science and technology | Conservative |
| 8744 | Post-80s | Female | Rural | Economic management | Adventurous |
| 8744 | Post-80s | Female | Rural | Economic management | Adventurous |
| 8744 | Post-80s | Male | Rural | Other types | Adventurous |
| 8744 | Post-90s | Male | Rural | Science and technology | Conservative |
| 8744 | Others | Female | Rural | Economic management | Intermediate |
| 8744 | Post-80s | Male | Rural | Science and technology | Intermediate |
| 8885 | Post-90s | Male | Rural | Science and technology | Conservative |
| 8885 | Post-90s | Male | Rural | Other types | Conservative |
| 8885 | Others | Female | Urban | Other types | Conservative |
| 9081 | Post-80s | Female | Rural | Science and technology | Conservative |
| 9081 | Post-80s | Male | Rural | Other types | Conservative |
| 9081 | Others | Male | Urban | Other types | Intermediate |
| 9109 | Post-00s | Male | Rural | Science and technology | Conservative |
| 9109 | Post-80s | Male | Rural | Economic management | Adventurous |
| 9109 | Post-90s | Male | Urban | Economic management | Intermediate |
| 9109 | Post-80s | Female | Rural | Other types | Adventurous |
| 9109 | Post-90s | Male | Urban | Other types | Intermediate |
| 9227 | Post-90s | Male | Rural | Other types | Intermediate |
| 9227 | Post-80s | Female | Urban | Science and technology | Intermediate |
| 9227 | Post-80s | Male | Rural | Science and technology | Conservative |
| 9227 | Post-90s | Male | Rural | Economic management | Intermediate |
| 9227 | Post-80s | Male | Rural | Other types | Adventurous |
| 9326 | Post-90s | Female | Urban | Other types | Adventurous |
| 9326 | Post-80s | Male | Urban | Economic management | Adventurous |
| 9326 | Post-80s | Female | Urban | Other types | Adventurous |
| 9326 | Post-80s | Male | Rural | Science and technology | Intermediate |
| 9675 | Post-90s | Male | Rural | Economic management | Adventurous |
| 9675 | Post-90s | Male | Rural | Science and technology | Adventurous |
| 9675 | Post-80s | Female | Rural | Science and technology | Adventurous |
| 1064 | Post-90s | Male | Rural | Other types | Intermediate |
| 1064 | Others | Male | Rural | Other types | Intermediate |
| 1064 | Others | Female | Rural | Other types | Intermediate |
| 1064 | Post-80s | Female | Urban | Science and technology | Adventurous |
| 1333 | Post-90s | Male | Rural | Economic management | Intermediate |
| 1333 | Others | Male | Urban | Other types | Intermediate |
| 1333 | Post-90s | Female | Rural | Science and technology | Conservative |
| 1333 | Others | Male | Urban | Other types | Adventurous |
| 1333 | Others | Male | Urban | Science and technology | Conservative |
| 1677 | Others | Female | Rural | Economic management | Intermediate |
| 1677 | Post-90s | Male | Rural | Other types | Conservative |
| 1677 | Post-90s | Male | Rural | Economic management | Intermediate |
| 1677 | Others | Female | Rural | Other types | Intermediate |
| 1677 | Post-80s | Male | Rural | Other types | Conservative |
| 1876 | Post-80s | Male | Rural | Science and technology | Adventurous |
| 1876 | Post-80s | Male | Rural | Economic management | Adventurous |
| 1876 | Post-80s | Male | Urban | Other types | Adventurous |
| 1876 | Post-90s | Male | Rural | Other types | Intermediate |
| 1876 | Post-90s | Male | Rural | Science and technology | Intermediate |
| 1987 | Post-90s | Male | Urban | Other types | Intermediate |
| 1987 | Others | Male | Rural | Science and technology | Intermediate |
| 1987 | Post-90s | Female | Rural | Science and technology | Adventurous |
| 1987 | Others | Female | Rural | Science and technology | Conservative |
| 2854 | Post-90s | Male | Rural | Economic management | Conservative |
| 2854 | Post-80s | Female | Rural | Other types | Adventurous |
| 2854 | Post-80s | Male | Urban | Science and technology | Intermediate |
| 2854 | Post-80s | Male | Rural | Other types | Intermediate |
| 2980 | Post-90s | Male | Urban | Science and technology | Conservative |
| 2980 | Post-00s | Male | Urban | Other types | Conservative |
| 2980 | Post-80s | Female | Urban | Science and technology | Intermediate |
| 3300 | Post-80s | Male | Urban | Economic management | Adventurous |
| 3300 | Post-90s | Male | Urban | Other types | Intermediate |
| 3300 | Post-80s | Female | Urban | Other types | Intermediate |
| 3314 | Post-80s | Male | Urban | Other types | Adventurous |
| 3314 | Post-80s | Female | Urban | Other types | Adventurous |
| 3314 | Others | Male | Urban | Other types | Conservative |
| 3314 | Post-80s | Male | Rural | Economic management | Intermediate |
| 3314 | Post-80s | Male | Rural | Economic management | Intermediate |
| 4159 | Post-80s | Female | Rural | Science and technology | Conservative |
| 4159 | Others | Male | Rural | Economic management | Intermediate |
| 4159 | Others | Male | Urban | Other types | Adventurous |
| 4159 | Post-80s | Male | Rural | Science and technology | Conservative |
| 4159 | Post-80s | Male | Rural | Science and technology | Intermediate |
| 4159 | Post-80s | Male | Rural | Other types | Intermediate |
| 4159 | Post-80s | Male | Rural | Other types | Intermediate |
| 4159 | Post-80s | Female | Rural | Economic management | Adventurous |
| 4231 | Post-90s | Male | Rural | Economic management | Adventurous |
| 4231 | Post-80s | Male | Urban | Economic management | Intermediate |
| 4231 | Post-80s | Male | Rural | Science and technology | Intermediate |
| 4231 | Others | Female | Rural | Other types | Conservative |
| 4440 | Post-90s | Female | Rural | Science and technology | Adventurous |
| 4440 | Others | Male | Urban | Other types | Intermediate |
| 4440 | Post-90s | Female | Rural | Other types | Intermediate |
| 4440 | Post-90s | Male | Rural | Other types | Intermediate |
| 4440 | Others | Female | Rural | Other types | Conservative |
| 4440 | Post-90s | Male | Rural | Economic management | Adventurous |
| 4440 | Post-80s | Male | Urban | Economic management | Intermediate |
| 4440 | Post-90s | Male | Urban | Other types | Adventurous |
| 4817 | Others | Male | Rural | Other types | Conservative |
| 4817 | Post-90s | Male | Urban | Science and technology | Intermediate |
| 4817 | Post-80s | Male | Rural | Economic management | Intermediate |
| 4817 | Others | Male | Rural | Science and technology | Conservative |
| 4983 | Others | Male | Urban | Other types | Intermediate |
| 4983 | Post-80s | Male | Rural | Science and technology | Conservative |
| 4983 | Post-90s | Female | Rural | Economic management | Adventurous |
| 4983 | Post-80s | Male | Urban | Science and technology | Conservative |
| 4983 | Post-80s | Male | Rural | Science and technology | Intermediate |
| 5710 | Others | Female | Rural | Science and technology | Intermediate |
| 5710 | Post-80s | Male | Urban | Other types | Conservative |
| 5710 | Others | Female | Rural | Other types | Adventurous |
| 5943 | Post-80s | Male | Rural | Science and technology | Intermediate |
| 5943 | Post-90s | Male | Urban | Economic management | Conservative |
| 5943 | Post-80s | Female | Rural | Other types | Intermediate |
| 5943 | Post-90s | Female | Rural | Science and technology | Adventurous |
| 6604 | Post-80s | Male | Rural | Other types | Conservative |
| 6604 | Post-90s | Male | Rural | Science and technology | Adventurous |
| 6604 | Post-90s | Male | Rural | Other types | Adventurous |
| 6604 | Others | Female | Urban | Other types | Intermediate |
| 6604 | Post-90s | Male | Rural | Science and technology | Conservative |
| 6604 | Post-80s | Female | Rural | Science and technology | Conservative |
| 6604 | Others | Female | Urban | Science and technology | Adventurous |
| 6729 | Others | Male | Urban | Economic management | Conservative |
| 6729 | Post-80s | Male | Urban | Other types | Conservative |
| 6729 | Post-90s | Male | Urban | Economic management | Conservative |
| 6729 | Others | Female | Urban | Other types | Adventurous |
| 7235 | Post-80s | Male | Rural | Other types | Intermediate |
| 7235 | Post-90s | Male | Rural | Science and technology | Adventurous |
| 7235 | Post-80s | Female | Rural | Science and technology | Conservative |
| 7440 | Post-90s | Male | Urban | Economic management | Intermediate |
| 7440 | Post-80s | Female | Urban | Other types | Intermediate |
| 7440 | Post-80s | Male | Urban | Science and technology | Conservative |
| 8160 | Post-80s | Male | Urban | Economic management | Conservative |
| 8160 | Post-80s | Male | Urban | Other types | Conservative |
| 8160 | Post-90s | Female | Rural | Science and technology | Intermediate |
| 8160 | Others | Male | Rural | Other types | Adventurous |
| 8483 | Post-90s | Male | Rural | Other types | Conservative |
| 8483 | Post-90s | Male | Urban | Science and technology | Adventurous |
| 8483 | Post-80s | Male | Urban | Other types | Conservative |
| 8483 | Others | Male | Urban | Other types | Adventurous |
| 8530 | Post-80s | Male | Urban | Economic management | Intermediate |
| 8530 | Others | Male | Urban | Science and technology | Conservative |
| 8530 | Post-80s | Female | Urban | Science and technology | Conservative |
| 8530 | Others | Male | Urban | Other types | Adventurous |
| 8530 | Post-80s | Male | Rural | Science and technology | Intermediate |
| 8530 | Post-80s | Male | Urban | Other types | Intermediate |
| 8530 | Post-80s | Male | Rural | Science and technology | Intermediate |
| 8583 | Post-90s | Male | Urban | Other types | Adventurous |
| 8583 | Post-80s | Female | Urban | Other types | Intermediate |
| 8583 | Post-80s | Male | Rural | Science and technology | Intermediate |
| 8583 | Post-90s | Male | Urban | Economic management | Intermediate |
| 8583 | Post-80s | Female | Rural | Science and technology | Intermediate |
| 8739 | Post-90s | Male | Rural | Other types | Conservative |
| 8739 | Post-90s | Male | Urban | Other types | Intermediate |
| 8739 | Post-80s | Female | Rural | Science and technology | Conservative |
| 8739 | Post-90s | Male | Rural | Economic management | Adventurous |
| 8744 | Post-80s | Male | Rural | Science and technology | Conservative |
| 8744 | Post-80s | Female | Rural | Economic management | Adventurous |
| 8744 | Post-80s | Female | Rural | Economic management | Adventurous |
| 8744 | Post-80s | Male | Rural | Other types | Adventurous |
| 8744 | Post-90s | Male | Rural | Science and technology | Conservative |
| 8744 | Others | Female | Rural | Economic management | Intermediate |
| 8744 | Post-80s | Male | Rural | Science and technology | Intermediate |
| 8885 | Post-90s | Male | Rural | Science and technology | Conservative |
| 8885 | Post-90s | Male | Rural | Other types | Conservative |
| 8885 | Others | Female | Urban | Other types | Conservative |
| 9081 | Post-80s | Female | Rural | Science and technology | Conservative |
| 9081 | Post-80s | Male | Rural | Other types | Conservative |
| 9081 | Others | Male | Urban | Other types | Intermediate |
| 9109 | Post-00s | Male | Rural | Science and technology | Conservative |
| 9109 | Post-80s | Male | Rural | Economic management | Adventurous |
| 9109 | Post-90s | Male | Urban | Economic management | Intermediate |
| 9109 | Post-80s | Female | Rural | Other types | Adventurous |
| 9109 | Post-90s | Male | Urban | Other types | Intermediate |
| 9227 | Post-90s | Male | Rural | Other types | Intermediate |
| 9227 | Post-80s | Female | Urban | Science and technology | Intermediate |
| 9227 | Post-80s | Male | Rural | Science and technology | Conservative |
| 9227 | Post-90s | Male | Rural | Economic management | Intermediate |
| 9227 | Post-80s | Male | Rural | Other types | Adventurous |
| 9326 | Post-90s | Female | Urban | Other types | Adventurous |
| 9326 | Post-80s | Male | Urban | Economic management | Adventurous |
| 9326 | Post-80s | Female | Urban | Other types | Adventurous |
| 9326 | Post-80s | Male | Rural | Science and technology | Intermediate |
| 9675 | Post-90s | Male | Rural | Economic management | Adventurous |
| 9675 | Post-90s | Male | Rural | Science and technology | Adventurous |
| 9675 | Post-80s | Female | Rural | Science and technology | Adventurous |

# The Difference between the Faultline Categories and Faultline Configurations

It is very important for readers to comprehend and clearly distinguish the two different concepts of the categories (or the dimensions) of the team faultlines and the configurations (or the combinations) of the team faultlines. In order to efficiently distinguish these two concept, the detailed explanations have been made as follows:

(1) The categories (or the dimensions) of the faultlines include 3 types: the information-decision faultlines, the background-experience faultlines, and the role-motivation faultlines. Each of the demographic faultline, like the gender faultline or expertise faultline, etc., belongs to different faultline categories, as the faultlines in the same category have similar natures, functions, or mechanisms to influence the entrepreneurial performance. They do not have any interactions and comprehensive effect at present, which means the value of the categoriesis to explain their common natures. The relevant descriptions have been added in the first paragraph in Section 2.2 in the new version of the manuscript. In addition, the empirical PCA and factor analysis are just used to confirm our summary in Literature Review Section, and select the most representative faultlines in each category for the further fsQCA (in Section 3.4), which is actually not a part of the main fsQCA.

(2) The configurations (or the combinations) of the faultlines include 4 types: Background-experience actuation; Guidance-balance lacking; Role-cognition conflict; Information-decision polarization, which are the main results of our fsQCA. A “configuration” is a combination of the ascendant conditions that interact with each other, forming an integrated effect on the final outcome. Specifically, in this study, a faultline configuration means several faultlines that may belong to different categories and occur in the same team, and they will interact with each other and have an overall influence to the final performance of the team. Mixed categories of faultlines may occur in the same team as a configuration, and affect the performance differently according to the mechanisms of different categories. In fact, as you can see in Table 6, we have totally 6 configurations. We summarize these configurations by their similarities of the faultlines within the configurations, so finally 4 types of configurations are found in our studies.

(3) Let’s take a more vivid example to understand it more clearly: Now you have 2 “categories” of foods, the “vegetables” and the “meats”. In these 2 categories, there are several specific foods, e.g., potatoes and tomatoes belong to the “vegetables”, while pork and beef belong to the “meat” (categorize the foods with same nature and attributes into the same type). Then you have the chances to cook them in the same dish, and that is how you construct the “configurations”, e.g., you can cook beef and potatoes in the same dish, or beef and tomatoes, or pork and tomatoes, or pork and potatoes. You can even try something strange or complex, like cooking beef and pork together, or beef, tomatoes and potatoes together, or all of them together if you like. Those dishes you cook can be seen as the different configurations: Although there may be sometimes “meats” and “vegetables” together, or all “meats”, or all “vegetables” (3 types of configurations, including several configurations mentioned before), they finally interact with each other in the same dish and have an integrated effect on the dish’s flavour. In other words, it is not the “categories” that decide the final dish, but the “configurations”. However, the categories of the foods can help us to understand the nutrient mechanism of the dish (configuration). I hope this example could be helpful to make you comprehend the question “why there are 3 categories of faultlines, but 4 types of configurations (including 6 configurations actually) occur in the result with incorporation” more easily, as well as to understand the different usages of them. Besides, the configurations in Table 6 are selected by the fsqca 3.0, which indicates these configurations are just the most significant (with a high coverage) ones in all of the configurations.

(4) To sum up, our logic about the categories and configurations are as follows: Firstly, we summarize the 3 different categories of the faultlines in order to learn how different types of faultlines work in the teams. Then, using PCA and factor analysis, we extract the most suitable faultlines to import into our further fsQCA study, which is the core part of our paper. Finally, 4 outstanding configurations with various categories of faultlines are found, and we explain why and how these configurations may totally affect the performance according to the interaction among the mechanisms of different categories of faultlines. And our discussion is based on the 4 configurations rather than the 3 categories.
